# Gut microbiota affects the activation of STING pathway and thus participates in the progression of colorectal cancer

**DOI:** 10.1186/s12957-024-03487-2

**Published:** 2024-07-25

**Authors:** Xinqiang Liu, Shasha Cui, Lu Zhang, Sainan Wu, Cunzhi Feng, Baozhi Liu, Huanlian Yang

**Affiliations:** 1 Department of Oncology, Binzhou People’s Hospital, First Ward, No.515, Huanghe 7th Road, Binzhou, Shandong Province 256600 PR China; 2 Department of Laboratory Medicine, Binzhou People’s Hospital, Binzhou, Shandong Province 256600 PR China; 3 General Surgery Department, Binzhou People’s Hospital, Binzhou, Shandong Province 256600 PR China

**Keywords:** Gut microbiota, Colorectal cancer, STING, 16S sequencing

## Abstract

**Background:**

More and more studies showed that gut microbiota was closely related to the development of colorectal cancer (CRC). However, the specific pathway of gut microbiota regulating CRC development is still unknown.

**Methods:**

We collected fecal samples from 14 CRC patients and 20 normal volunteers for 16 S sequencing analysis. At the same time, 14 CRC patients’ tumors and their adjacent tissues were collected for the detection of STING pathway related protein level. Mice were injected with azoxymethane (AOM) to establish an animal model of CRC, and antibiotics were given at the same time to evaluate the influence of gut microbiota on STING pathway and whether it was involved in regulating the tumor development of CRC mice.

**Results:**

The sequencing results showed that compared with the normal group, the gut microbiota gut microbiota of CRC patients changed significantly at different species classification levels. At the level of genus, *Akkermansia*,* Ligilactobacillus* and *Subdoligranulum* increased the most in CRC patients, while *Bacteroides* and *Dialister* decreased sharply. The expression of STING-related protein was significantly down-regulated in CRC tumor tissues. Antibiotic treatment of CRC mice can promote the development of tumor and inhibit the activation of STING pathway.

**Conclusion:**

Gut microbiota participates in CRC progress by mediating STING pathway activation.

**Supplementary Information:**

The online version contains supplementary material available at 10.1186/s12957-024-03487-2.

## Highlights

**•** 16 S sequencing was used to analyze the gut microbiota in the feces of CRC patients.
**•** STING pathway protein was down-regulated in tumor tissues of CRC patients.
**•** STING pathway mediated by gut microbiota affects tumor development in CRC mice.

**Supplementary Information:**

The online version contains supplementary material available at 10.1186/s12957-024-03487-2.

## Introduction

Colorectal cancer (CRC) is the fourth most deadly cancer in the world, with nearly 900,000 colorectal cancer patients dying every year [1]. With the aging of population in more and more countries, the increase of unfavorable risk factors such as changing eating habits and lack of physical exercise will also increase the risk of CRC [2]. At present, the routine treatment of CRC includes surgery, chemotherapy and radiotherapy. And according to the disease progress of CRC patients, different treatment methods can be combined [3]. However, it is difficult to completely remove all cancer cells in patients with metastatic colorectal cancer [4]. Chemotherapy and radiotherapy have strong side effects [5]. In recent years, cancer immunotherapy is also one of the new options for CRC treatment [6]. More and more researchers focus on the in-depth study of CRC progression mechanism, which will help us identify new biomarkers and improve treatment.

In recent years, the research on CRC has found that the human gut microbiota is closely related to the occurrence and development of CRC [7]. Gut microbiota is a large of microorganisms that interact with host cells near colorectal epithelium, and they mainly participate in energy metabolism and immune response of colorectal cells [8]. Recently, more and more studies have found that the changes of gut microbiota composition and metabolites of cancer patients cause the differential expression of oncogenes in host cells, the change of immune microenvironment and the imbalance of metabolic balance [9]. Related studies have analyzed the gut microbiota in the feces of CRC patients, and confirmed that many bacteria such as *Fusobacterium nucleatum* and *Enterococcus faecalis* may be involved in the occurrence of colorectal cancer [10]. Further functional studies in animal models showed that metabolites such as short-chain fatty acids and taurodeoxycholic acid played a key role in the development of CRC [11, 12]. These studies will lay a theoretical foundation for the clinical treatment of CRC by using gut microbiota. Because the relationship between gut microbiota and CRC is not clear, the related mechanism needs to be further explored.

The Stimulator of interferon genes (STING) is a pathway involved in innate immunity [13]. STING agonists have shown anti-cancer effects in related clinical studies and experiments [14]. Activating STING signaling can promote the anti-PD-L1 immunotherapy of CRC [15]. In mice with peritoneal metastasis of CRC, intraperitoneal injection of STING agonist can normalize the peritoneal immune microenvironment of mice and prevent peritoneal spread of CRC cells [16]. Interestingly, in one study, gut microbiota promoted the host’s ability to resist viral infection through STING signaling pathway [17]. In tumor research, it has been confirmed that gut microbiota promotes anti-CD47 mediated immunotherapy by activating STING pathway [18]. However, it has not been reported whether the changes of gut microbiota in CRC participate in the progress of colon cancer through STING. We will further explore the relationship between the changes of gut microbiota in CRC and STING.

In this study, the changes of gut microbiota structure will affect the activation of STING pathway in cells and participate in CRC progress in patients with CRC and children with CRC. The structural differences of gut microbiota in clinical level were determined by 16s sequencing of feces of CRC patients and detection of pathway proteins in tumor tissues. In the CRC mouse model, it was verified that gut microbiota affected STING pathway and participated in the tumor progression of CRC mice.

## Methods

### Collection and treatment of clinical tissues and feces of CRC patients

The tumor and adjacent tissue samples of 14 patients with colorectal cancer who were clinically diagnosed and underwent surgical resection in Binzhou People’s Hospital were collected. Tissue samples were stored at -80℃. 20 g fecal samples were collected from 14 CRC patients and 20 healthy volunteers, which were stored at 4℃ before analysis. All the patients and volunteers are Han people in Shandong province, with similar geographical areas and eating habits. Exclusion criteria are as follows: (1) Patients over 80 years old or under 25 years old; (2) Patients with familial colorectal cancer and inflammatory bowel disease (IBD); (3) Have a history of other malignant tumors or are receiving anti-tumor treatment; (4) Antibiotics, probiotics, prebiotics, hormones, steroids or synbiotics were used 6 months before fecal sample collection. All volunteers were between 30 and 70 years old, and the sex ratio was consistent with that of CRC patients. Volunteers had normal defecation habits and no other major diseases. The suspension of feces was homogenized by a homogenizer (FastPrep 24, MP Biomedi, USA). Then, the fecal DNA extraction kit (116,570,200, MP Biomedi, USA) was used to extract DNA from the fecal samples.

### Sequencing and analysis of 16 S intestinal flora

16 S rRNA gene (319 F: 5′-ACTCCTACGGGAGGCAGCAG-3′; 806R: 5′-GGACTACHVGGGTWTCTAAT-3′) of bacteria in feces was amplified and sequenced, and the difference of gut microbiota composition was evaluated [19]. Triplicates were pooled, and the PCR amplicons were sequenced using an Illumina HiSeq platform (Illumina MiSeq, USA). The sequencing results were processed by QIIME2 platform (v2021.4). The original data were filtered by using the DADA2 plug-in, and the amplicon sequence variants (ASVs) table was constructed. Then, the VSEARCH plug-in is used to cluster ASVs, and those with more similarity than 97% belong to the same operational classification unit (OTUs). According to the 16 S rRNA reference of RDP, mothur20 was used to classify the representative sequences of each OTUs. Gut microbiota α-diversity was analyzed according to OUT information using QIIME software. β-diversity was estimated using Bray-Curtis dissimilarity and the Jaccard similarity index between samples. Bacterial diversity was expressed by observed ASVs count and Shannon index. Principal co-ordinates analysis (PCoA) and partial least square discriminant analysis (PLS-DA) were utilized to evaluate global microbiota composition (β-diversity) based on Bray-Curtis distances with statistical differences between groups calculated by analysis of similarities (ANOISM) and permutational multivariate analysis of variance (PERMANOVA).

### Construction of CRC mouse model

C57BL/6 male mice (6–8 weeks old) were purchased from Hunan SJA (Hunan SJA Laboratory Animal Co., Changsha, China). According to the previous research method [20], mice were intraperitoneally injected with 10 mg/kg of azoxymethane (AOM, A5486-25MG, Sigma-Aldrich, USA) every day for seven days. Then, mice were fed with 2% (weight/volume) dextran sodium sulfate (DSS, PHL 83,846, Sigma-Aldrich) for 7 consecutive days. Subsequently, normal drinking water feeding was resumed for 14 consecutive days. This feeding cycle was repeated twice continuously until the 70th day, and the mice were killed by dislocation of the spine and their tumors were collected. A mixture containing Vancomycin, Neomycin and Ampicillin (1 mg/mL) was administered to antibiotic treatment group [21]. The antibiotic mixture was added to the drinking water at age 10 and 14 weeks. Normal drinking water replaced the antibiotic containing water after 10 days of administration. All animal experiments were approved by the Ethics Approval Centre of Medical Ethics Committee of Binzhou People’s Hospital (2020266).

### Western blot

Firstly, the tumor tissue was homogenized by a homogenizer, and then the tumor tissue was lysed by RIPA lysis buffer (#20–188, Merck Millipore, USA). Protein was separated by SDS-polyacrylamide gel, and the blot was transferred to a PVDF membrane (Merck Millipore) and sealed with 5% dehydrated milk. The membrane was incubated with primary antibody at 4℃ overnight. After washing the membrane with TBS for three times, the membrane was incubated with HRP-linked secondary antibody for 1 h at room temperature. β-actin was used as internal control. Finally, the protein bands were treated by enhanced chemiluminescence kit (Millipore, USA). Primary antibodies: anti-cGAS (1:1000, PA5-121188, Thermo Fisher Scientific, China), anti-p-STING (1: 1000, PA5-105674, Thermo Fisher Scientific, China), anti-STING (1:1000, PA5-23381, Thermo Fisher Scientific, China), anti-p-TBK1 (1:1000, PA5-105919, Thermo Fisher Scientific, China), anti-TBK1 (1:5000, ab40676, Abcam, UK), anti-p-IRF3 (1:1000, PA5-38285, Thermo Fisher Scientific, China), anti-IRF3 (1:1000, ab68481, Abcam, UK) and anti-β-actin (1:1000, ab8226,Abcam, UK).

### Immunohistochemistry

Mouse colon tumor tissue was fixed with formaldehyde and then embedded in paraffin, and then made into sections. Then, after dewaxing, the slices were antigen-repaired in citrate buffer (pH 6.0). The slices were sealed at room temperature in 0.3% H_2_O_2_ and normal goat serum. The primary antibody incubated the slices at 4℃ overnight. Subsequently, the target protein was stained with streptavidin-peroxidase (A9044, Sigma-Aldrich) and labeled with 3,3’-diaminobenzidine (D12384, Sigma-Aldrich). Related protein primary antibody: anti-cGAS (1:1000, PA5-121188, Thermo Fisher Scientific, China), anti-p-STING (1:1000, PA5-105674, Thermo Fisher Scientific, China), Ki67 (1:5000, ab15580, Abcam, UK).

### Statistical analysis

All experimental results are expressed as mean standard deviation (SD). Graph Pad Prism 8 was used for statistical analysis. Hierarchical clustering analysis was applied on Pearson distances using PermutMatrix. Two-tailed Student’s t-test was performed and the p-value was adjusted by the Benjamini-Hochberg (BH) correction. The probability level for statistical tests was set at α = 0.05 and was adjusted by the BH correction to allow for a maximum 5% probability (q = 0.05) of false positive detection. ANOVA was used to compare differences among multiple groups, and post hoc analysis was performed by Tukey’s multiple comparisons test. P value < 0.05 indicates statistical significance.

## Results

### 16 S sequencing analysis of fecal samples from patients with colorectal cancer and healthy volunteers

**Fig. 1:**
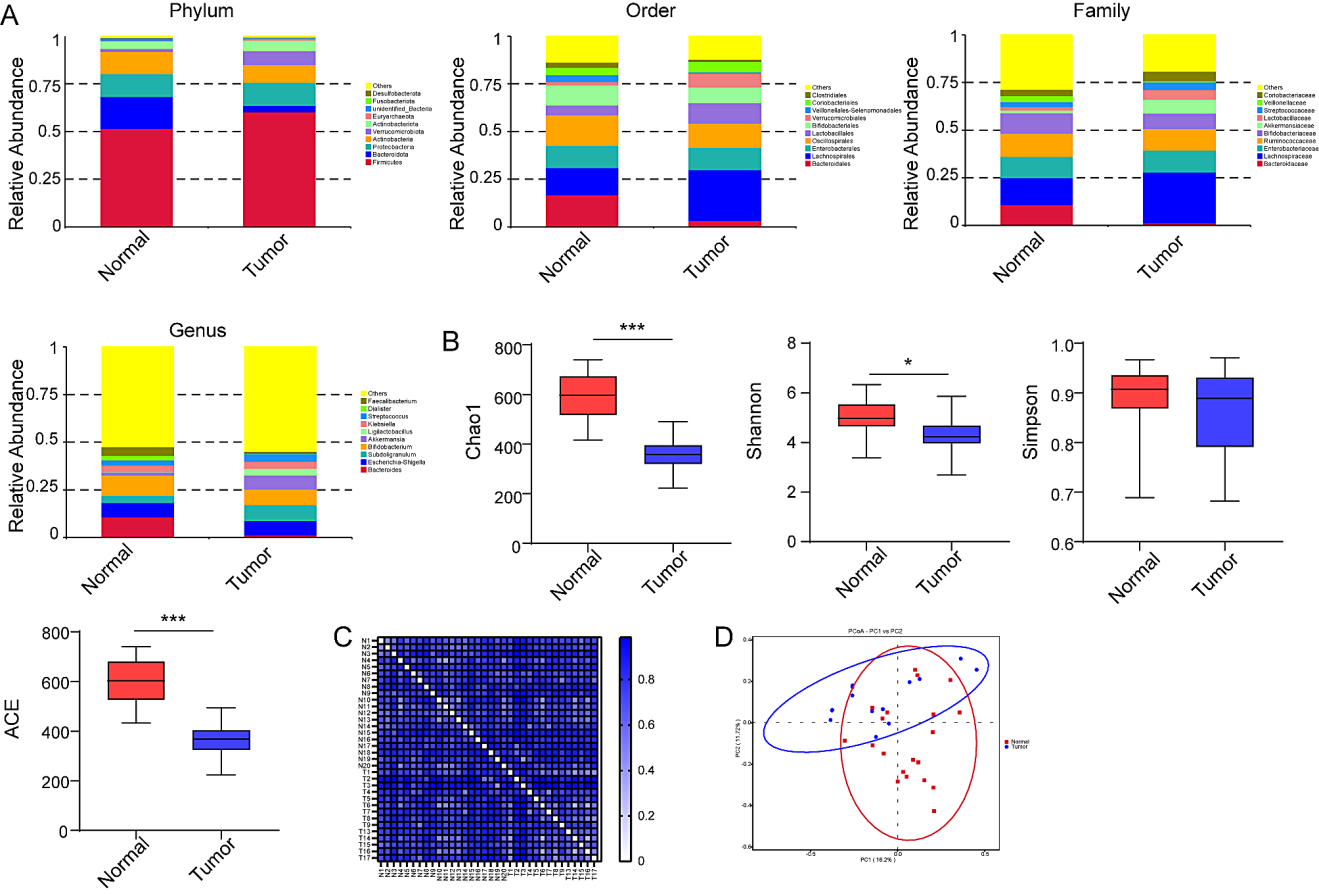
16 S sequencing analysis of fecal samples from patients with colorectal cancer and healthy volunteers. (**A**) The relative abundance map of gut microbiota at the levels of phylum, order, family and genus in normal group (*n* = 20) and tumor group (*n* = 14). (**B**) The changes of α diversity in normal group and tumor group include Chao1, Shannon, Simpson and ACE index. (**C**) Matrix diagram of distance between samples. (**D**) PCoA analysis chart of β diversity

In order to explore the difference of gut microbiota between CRC patients and healthy volunteers, we analyzed the results based on 16 S rRNA gene sequencing of fecal samples. The gut microbiota of CRC patients had changed at different classification levels (Fig. 1A). At the phylum level, *Firmicutes* was dominant. Compared with the normal group, the changes of *Bacteroidota* in tumor patients were the biggest. At the order level, the *Bacteroidales* and *Coriobacteriales* in gut microbiota of tumor patients decreased significantly, while *Lachnospirales*, *Lactobacillales* and *Verrucomicrobiales* increased remarkably. Further analysis at the family level showed that the abundance of *Lachnospiraceae*, *Lactobacillaceae* and *Akkermansiaceae* increased, while the proportion of *Bacteroidaceae* decreased most notably. Finally, at the genus level, *Akkermansia*, *Ligilactobacillus* and *Subdoligranulum* increased the most in tumor patients, while *Bacteroides* and *Dialister* decreased dramatically. This showed that there were differences in the abundance of gut microbiota in CRC patients at different species classification levels, and the proportion of species in each group has changed noticeably.

Then, we analyzed the α diversity of microorganisms in CRC patients. Chao1, Shannon, Simpson and ACE decreased in Tumor group, and Simpson decreased with no significant difference, while others showed significant differences. It is suggested that the total number of species, community diversity and evenness decreased significantly (Fig. 1B). The distance matrix diagram between samples and PCoA analysis showed that there were some differences in community structure between normal group and tumor group (Fig. 1C and D). The results showed that the gut microbiota of CRC patients changed significantly.

**Fig. 2:**
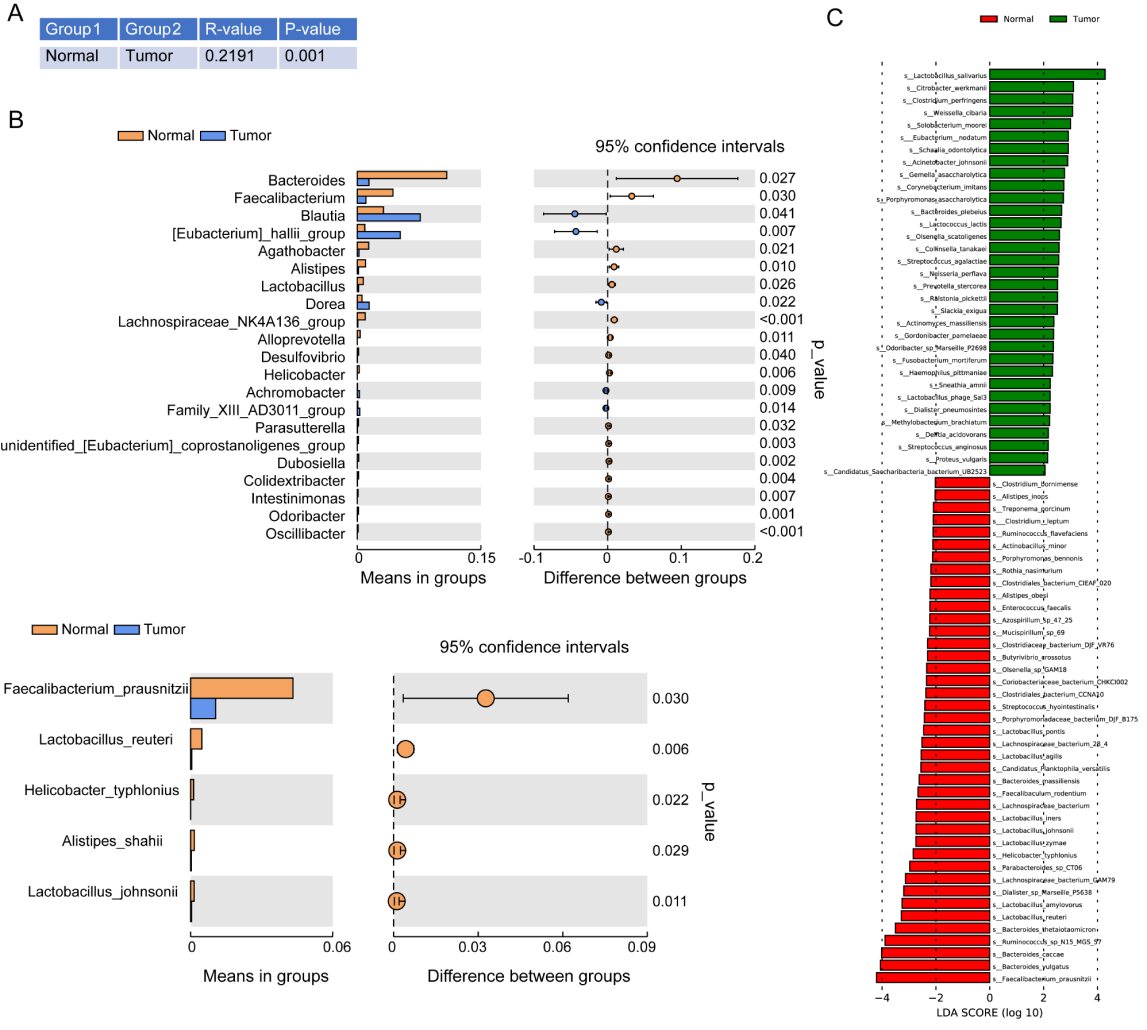
16 S sequencing analysis of fecal samples from patients with colorectal cancer and healthy volunteers. (**A**) ANOISM analysis of β difference between normal group (*n* = 20) and tumor group (*n* = 14) (*R* = 0.2191, *p* < 0.05). (**B**) T-test analysis was used to screen the different species at the level of microbial species and genera. (**C**) obtaining each group of markers based on LEfSe

Furthermore, we analyzed ANOISM, which represents the β diversity between normal group and tumor group, and the analysis showed that there were differences between the two groups (*R* > 0, *p* < 0.05, Fig. 2A). We screened out the different species at the genus and species levels of the two groups of samples. 21 microbial genus showed obvious relative abundance between normal group and tumor group, and 5 microbial species showed different abundance between normal group and tumor group (Fig. 2B). Through linear discriminant analysis and effect size analysis (LEfSe), we found that 74 microbial groups showed obvious relative abundance between normal group and tumor group (LDA score > 2.0, *p* < 0.05, Fig. 2C). The above results showed that the statistical distribution and relative abundance changes of microbial markers in CRC patients’ samples.

### Difference of STING protein pathway in CRC clinical tissue samples

**Fig. 3:**
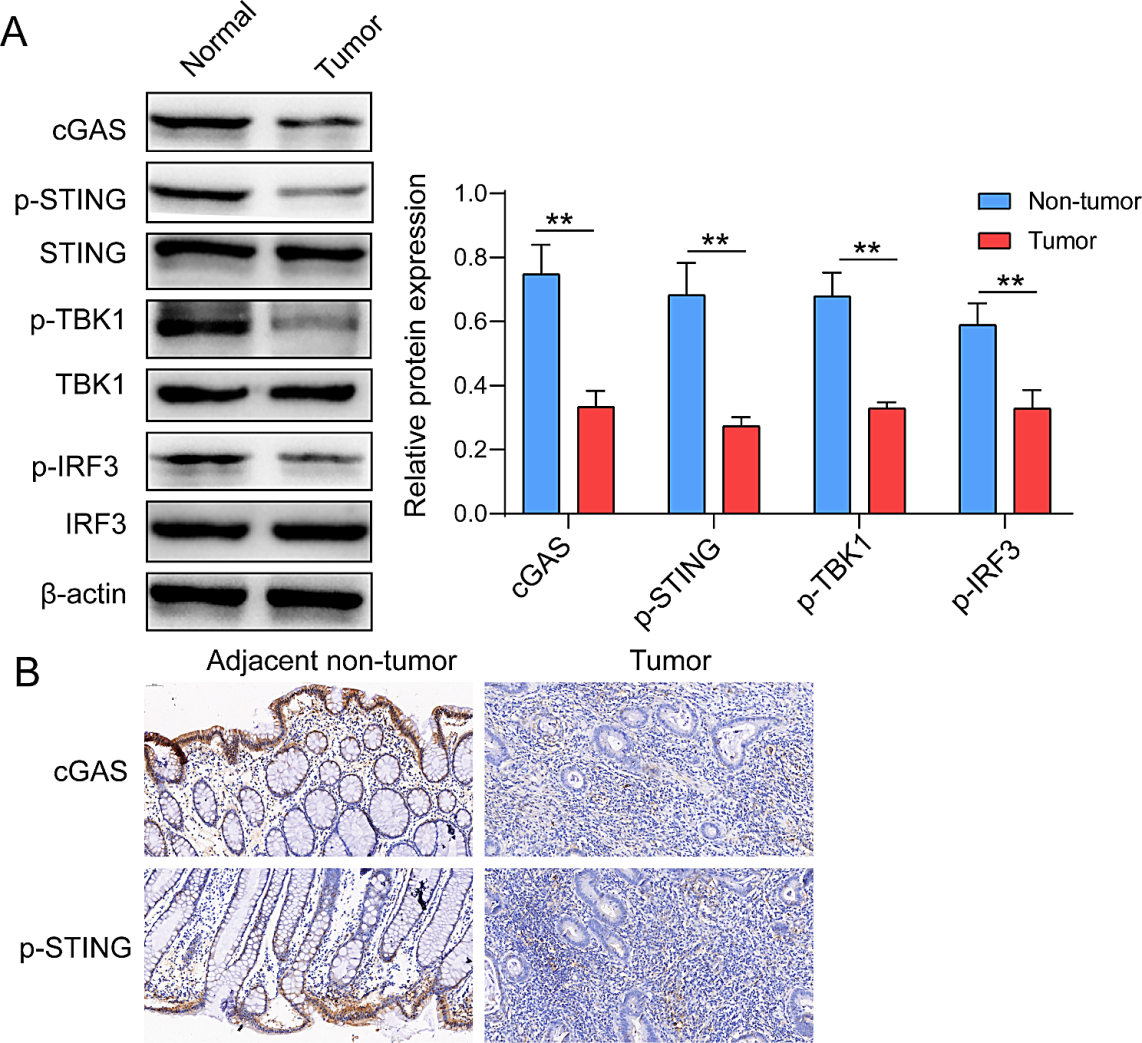
Difference of STING protein pathway in CRC clinical tissue samples. (**A**) The protein levels of cGAS, p-STING, STING, p-TBK1, TBK1, p-IRF3 and IRF3 in CRC tissues were detected by western blot. (**B**) The levels of cGAS and p-STING protein in CRC tissues were detected by immunohistochemistry. **p < 0.01

The differences of STING pathway were evaluated by collecting tumor and adjacent tissues of CRC patients and detecting the related protein expression level. Results as shown in Fig. 3A, the protein expression levels of cGAS and phosphorylation levels of STING, TBK1 and IRF3 in tumor tissues were significantly lower than those in adjacent tissues (Fig. 3A). Further, it was found by immunohistochemical detection that the expression of cGAS protein and the level of STING phosphorylation in tumor tissues were remarkable decreased compared with those in adjacent tissues (Fig. 3B). Therefore, it was suggested that the change of STING pathway protein is of significance to the development of CRC.

### Gut microbiota affects STING pathway and participates in tumor progression in CRC mice

**Fig. 4:**
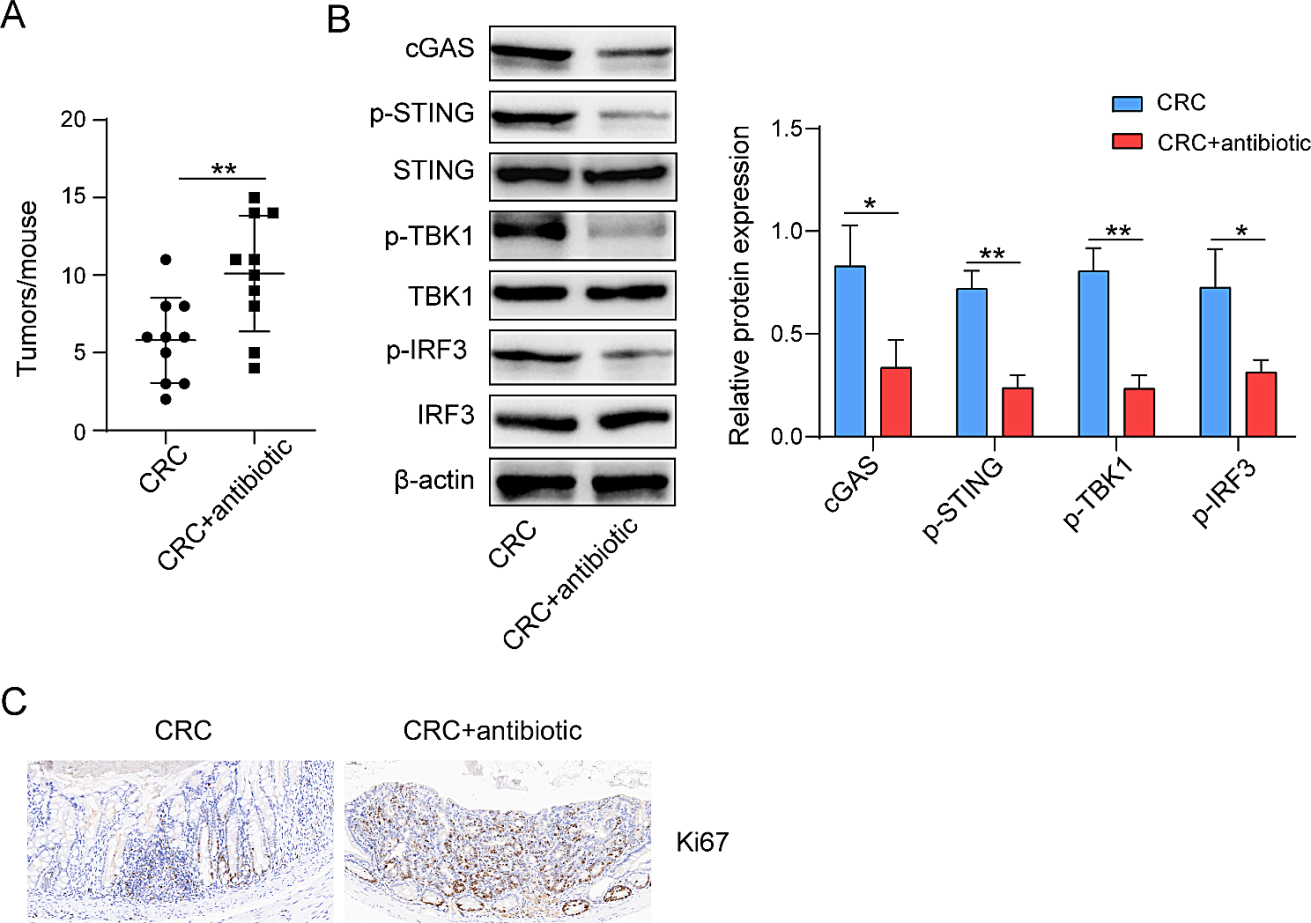
Gut microbiota affects STING pathway and participates in tumor progression in CRC mice The CRC mice were treated by oral antibiotics. (**A**) After collecting mice’s colorectal cancer, the number of tumors were counted. (**B**) The protein levels of cGAS, p-STING, STING, p-TBK1, TBK1, p-IRF3 and IRF3 in mice CRC tissues were detected by western blot. (**C**) The levels of Ki67 protein in mice CRC tissues were detected by immunohistochemistry. *p < 0.05, **p < 0.01

Finally, we conducted antibiotic therapy on CRC to verify that gut microbiota participated in tumor progression of CRC mice through STING pathway. The colorectal tissues of experimental mice were collected, and it was found that compared with the model group, the number of tumors in the antibiotic group increased significantly (Fig. 4A). Through the detection of protein related to STING pathway, it was found that the protein expression levels of cGAS and phosphorylation levels of decreased significantly after antibiotic treatment (Fig. 4B). Finally, the level of Ki67 in colorectal tissues of mice treated with antibiotics increased dramatically (Fig. 4C). To sum up, gut microbiota might participate in the occurrence and development of CRC mice tumors by activating STING pathway.

## Discussion

With the change of people’s diet structure and the increase of obesity rate, the onset age of CRC is getting younger and the prognosis is poor [22]. At the same time, the gut microbiota is also influenced by age and diet [23]. In recent years, there have been more and more studies on the relationship between gut microbiota and cancer [24]. It was found that STING signal regulated by gut microbiota could increase anti-liver cancer radiotherapy [25]. Inspired by these studies, we put forward the hypothesis that gut microbiota participates in CRC progress by mediating STING pathway activation. Our hypothesis was confirmed by 16s sequencing of clinical samples of CRC patients and related animal experiments.

It was found that most CRC patients will have gut microbiota imbalance [26]. In addition, *bacteroides fragilis*, *Clostridium difficile* and *Streptococcus digestion* were the main microorganisms enriched in the intestines of CRC patients [27]. We collected the feces of CRC patients for 16s sequencing analysis, and found that *Akkermansia*,* Ligilactobacillus* and *Subdoligranulum* increased the most in CRC patients. *Akkermansia* was a Gram-negative anaerobic bacterium. In CRC, *Akkermansia* changed tumor immune microenvironment homeostasis by secreting acetyltransferase, thus inhibiting the proliferation of CRC [28]. An interesting study found that *Ligilactobacillus* might be the key driving factor in the carcinogenesis of CRC [29]. In our study, the enrichment of *Akkermansia*, *Ligilactobacillus* and *Subdoliglanum* in the intestine of CRC patients might participate in the regulation of CRC progress. Therefore, the growth of such microorganisms could be further regulated to inhibit the development of CRC. However, whether fecal microflora from CRC patients can also induce CRC metastasis needs further discussion. Moreover, the related metabolites of the strain are still unclear, and we need to continue our research.

CGAS-STING signaling pathway mediated anti-tumor immune response by inducing cytoplasmic DNA up-regulated type I interferon (IFNs) [30]. STING signal was the main regulator of colorectal homeostasis and cancer development. STING signal could promote the secretion of IL-1β and IL-18 in colorectal tissue, and IL-1β and IL-18 could prevent gut microbiota from activating inflammation by preventing intestinal injury, thus inhibiting the possibility of canceration [31]. Through the detection of proteins related to STING pathway in clinical tumor samples of CRC patients, we found that the levels of cGAS, STING, TBK1, IRF3 proteins and phosphorylation in CRC tissues all decreased. STING protein in intestine could respond to the stimulation of *lactic acid bacteria*, thus activating gut microbiota and regulating innate immune response [32]. We treated CRC mice with antibiotics to disturb the homeostasis of gut microbiota. Through the detection of proteins related to STING pathway in mouse tissues, it was found that STING pathway was inhibited after antibiotic treatment. Therefore, we speculate that gut microbiota could participate in the tumor progression of CRC mice by affecting STING pathway. However, our research also has some limitations. We did not inhibit the STING pathway to explore the effect of gut microbiota changes on STING activation. And it is impossible to determine which bacteria metabolites have a direct effect on STING pathway.

Our research is exploratory, which has some limitations in the demonstration of causality. Because the microorganisms in mice and their eating habits are not completely consistent with those of human fatigue, and there may be potential deviation between the evaluation of the influence of mice’s own intestinal flora on CRC and the results of clinical patients, we can explore it by transplanting human fatigue feces in the future. In addition, the intestinal feces of patients or volunteers are greatly influenced by eating habits and lifestyles. Because of the lack of diet control and the simplicity of study design, we can only associate metabolites with disease phenotypes at one time point in the two groups.

## Conclusions

In summary, our study identified significant differences in bacterial genera by clustering analysis of intestinal gut microbiota in CRC patients’ feces through 16s sequencing. Further, by detecting the changes of related proteins in CRC tumor tissues, it was found that there were significant differences in STING pathway related proteins. Finally, by constructing CRC mice and changing the homeostasis of gut microbiota in mice, it was found that gut microbiota participated in tumor progression in CRC mice through mediating STING pathway.

### Electronic supplementary material

Below is the link to the electronic supplementary material.

Supplementary Material 1

Supplementary Material 2

## Data Availability

No datasets were generated or analysed during the current study.

## Abbreviations

CRC: Colorectal cancer
STING: Stimulator of interferon genes
ASVs: Amplicon sequence variants
OTUs: Operational classification unit
AOM: Azoxymethane
DSS: Dextran sodium sulfate
IFNs: Interferons

## References

[1] Zhang ZW, Liu XH, Yang XQ, Jiang Y, Li A, Cong JY et al. Identification of faecal extracellular vesicles as novel biomarkers for the non-invasive diagnosis and prognosis of colorectal cancer. J Extracell Vesicles. 2023,12(1). 10.1002/jev2.12300 PMC9816085 36604402

[2] Van Cutsem E, Cervantes A, Adam R, Sobrero A, Van Krieken JH, Aderka D, et al. ESMO consensus guidelines for the management of patients with metastatic colorectal cancer. Ann Oncol. 2016;27(8):1386–422. 27380959 10.1093/annonc/mdw235

[3] Johdi NA, Sukor NF. Colorectal Cancer immunotherapy: options and strategies. Front Immunol. 2020,11. 10.3389/fimmu.2020.01624 PMC7530194 33042104

[4] Shin AE, Giancotti FG, Rustgi AK. Metastatic colorectal cancer: mechanisms and emerging therapeutics. Trends Pharmacol Sci. 2023;44(4):222–36. 36828759 10.1016/j.tips.2023.01.003 PMC10365888

[5] Garg MB, Lincz LF, Adler K, Scorgie FE, Ackland SP, Sakoff JA. Predicting 5-fluorouracil toxicity in colorectal cancer patients from peripheral blood cell telomere length: a multivariate analysis. Brit J Cancer. 2012;107(9):1525–33. 22990653 10.1038/bjc.2012.421 PMC3493765

[6] Gajewski TF, Schreiber H, Fu YX. Innate and adaptive immune cells in the tumor microenvironment. Nat Immunol. 2013;14(10):1014–22. 24048123 10.1038/ni.2703 PMC4118725

[7] Wong CC, Yu J. Gut microbiota in colorectal cancer development and therapy. Nat Rev Clin Oncol. 2023;20(7):429–52. 37169888 10.1038/s41571-023-00766-x

[8] Wong SH, Yu J. Gut microbiota in colorectal cancer: mechanisms of action and clinical applications. Nat Rev Gastro Hepat. 2019;16(11):690–704. 10.1038/s41575-019-0209-8 31554963

[9] Song M, Chan AT, Environmental, Factors. Gut microbiota, and Colorectal Cancer Prevention. Clin Gastroenterol H. 2019;17(2):275–89. 10.1016/j.cgh.2018.07.012 PMC6314893 30031175

[10] Gagnière J, Raisch J, Veziant J, Barnich N, Bonnet R, Buc E, et al. Gut microbiota imbalance and colorectal cancer. World J Gastroentero. 2016;22(2):501–18. 10.3748/wjg.v22.i2.501 PMC4716055 26811603

[11] Hou HQ, Chen DF, Zhang KX, Zhang WR, Liu TY, Dai X, et al. Gut microbiota-derived short-chain fatty acids and colorectal cancer: ready for clinical translation? Cancer Lett. 2022;526:225–35. 34843863 10.1016/j.canlet.2021.11.027

[12] Bai XW, Wei H, Liu WX, Coker OO, Gou HY, Liu CA, et al. Cigarette smoke promotes colorectal cancer through modulation of gut microbiota and related metabolites. Gut. 2022;71(12):2439–50. 35387878 10.1136/gutjnl-2021-325021 PMC9664112

[13] Lv J, Zhu XX, Xing CL, Chen YH, Bian HH, Yin H et al. Stimulator of interferon genes (STING): key therapeutic targets in ischemia/reperfusion injury. Biomed Pharmacother. 2023,167. 10.1016/j.biopha.2023.115458 37699319

[14] Flood BA, Higgs EF, Li SY, Luke JJ, Gajewski TF. STING pathway agonism as a cancer therapeutic. Immunol Rev. 2019;290(1):24–38. 31355488 10.1111/imr.12765 PMC6814203

[15] Liu CF, Wang X, Qin W, Tu JY, Li CY, Zhao WH, et al. Combining radiation and the ATR inhibitor berzosertib activates STING signaling and enhances immunotherapy via inhibiting SHP1 function in colorectal cancer. Cancer Commun. 2023;43(4):435–54. 10.1002/cac2.12412 PMC10091106 36855844

[16] Lee SJ, Yang H, Kim WR, Lee YS, Lee WS, Kong SJ et al. STING activation normalizes the intraperitoneal vascular-immune microenvironment and suppresses peritoneal carcinomatosis of colon cancer. J Immunother Cancer. 2021,9(6). 10.1136/jitc-2020-002195 PMC8215239 34145029

[17] Erttmann SF, Swacha P, Aung KM, Brindefalk B, Jiang H, Härtova A, et al. The gut microbiota prime systemic antiviral immunity via the cGAS-STING-IFN-I axis. Immunity. 2022;55(5):847–. 35545033 10.1016/j.immuni.2022.04.006

[18] Shi YY, Zheng WX, Yang KT, Harris KG, Ni KY, Xue L et al. Intratumoral accumulation of gut microbiota facilitates CD47-based immunotherapy via STING signaling. J Exp Med. 2020,217(5). 10.1084/jem.20192282 PMC7201921 32142585

[19] Sugimura N, Li Q, Chu ESH, Lau HCH, Fong W, Liu WX, et al. Modulates the gut microbiota and produces anti-cancer metabolites to protect against colorectal tumourigenesis. Gut. 2022;71(10):2011–21. 10.1136/gutjnl-2020-323951 PMC9484392 34937766

[20] Bao Y, Zhai JN, Chen HR, Wong CC, Liang C, Ding YQ, et al. Targeting m6A reader YTHDF1 augments antitumour immunity and boosts anti-PD-1 efficacy in colorectal cancer. Gut. 2023;72(8):1497–509. 36717220 10.1136/gutjnl-2022-328845 PMC10359538

[21] Kaur K, Saxena A, Debnath I, O’Brien JL, Ajami NJ, Auchtung TA, et al. Antibiotic-mediated bacteriome depletion in mice is associated with reduction in mucus-producing goblet cells and increased colorectal cancer progression. Cancer Med-Us. 2018;7(5):2003–12. 10.1002/cam4.1460 PMC5943478 29624892

[22] Thanikachalam K, Khan G. Colorectal Cancer Nutr Nutrients. 2019,11(1). 10.3390/nu11010164 PMC6357054 30646512

[23] Huttenhower C, Gevers D, Knight R, Abubucker S, Badger JH, Chinwalla AT, et al. Structure, function and diversity of the healthy human microbiome. Nature. 2012;486(7402):207–14. 22699609 10.1038/nature11234 PMC3564958

[24] Chrysostomou D, Roberts LA, Marchesi JR, Kinross JM. Gut microbiota modulation of efficacy and toxicity of Cancer Chemotherapy and Immunotherapy. Gastroenterology. 2023;164(2):198–213. 36309208 10.1053/j.gastro.2022.10.018

[25] Li ZJ, Zhang Y, Hong WF, Wang B, Chen YX, Yang P et al. Gut microbiota modulate radiotherapy-associated antitumor immune responses against hepatocellular carcinoma Via STING signaling. Gut Microbes. 2022,14(1). 10.1080/19490976.2022.2119055 PMC9467592 36093568

[26] Wang ZK, Dan WY, Zhang NA, Fang JY, Yang YS. Colorectal cancer and gut microbiota studies in China. Gut Microbes. 2023,15(1). 10.1080/19490976.2023.2236364 PMC10364665 37482657

[27] Li L, Li XF, Zhong WL, Yang M, Xu MQ, Sun Y, et al. Gut microbiota from colorectal cancer patients enhances the progression of intestinal adenoma in mice. Ebiomedicine. 2020;53:301. 10.1016/j.ebiom.2020.102680 PMC6838415 31594750

[28] Jiang Y, Xu YJ, Zheng C, Ye L, Jiang P, Malik S, et al. Acetyltransferase from blunts colorectal tumourigenesis by reprogramming tumour microenvironment. Gut. 2023;72(7):1308–18. 36754607 10.1136/gutjnl-2022-327853

[29] Wang Y, Zhang Y, Qian Y, Xie YH, Jiang SS, Kang ZR, et al. Alterations in the oral and gut microbiome of colorectal cancer patients and association with host clinical factors. Int J Cancer. 2021;149(4):925–35. 10.1002/ijc.33596 33844851

[30] Tian JR, Zhang DY, Kurbatov V, Wang QR, Wang YD, Fang D et al. 5-Fluorouracil efficacy requires anti-tumor immunity triggered by cancer-cell-intrinsic STING. Embo J. 2021,40(7). 10.15252/embj.2020106065 PMC8013832 33615517

[31] Hu QY, Zhou Q, Xia XF, Shao LH, Wang M, Lu XF et al. Cytosolic sensor STING in mucosal immunity: a master regulator of gut inflammation and carcinogenesis. J Exp Clin Canc Res. 2021,40(1). 10.1186/s13046-021-01850-9 PMC7825222 33485379

[32] Gutierrez-Merino J, Isla B, Combes T, Martinez-Estrada F, De Motes CM. Beneficial bacteria activate type-I interferon production via the intracellular cytosolic sensors STING and MAVS. Gut Microbes. 2020,11(4). 10.1080/19490976.2019.1707015 PMC7524384 31941397

