# Supplementary figures and images for: Gut microbiota affects the activation of STING pathway and thus participates in the progression of colorectal cancer
Source: World J Surg Oncol. 2024 Jul 25;22:192. doi: 10.1186/s12957-024-03487-2 (PMC11270765; doi:10.1186/s12957-024-03487-2)

Fig3 A

cGAS


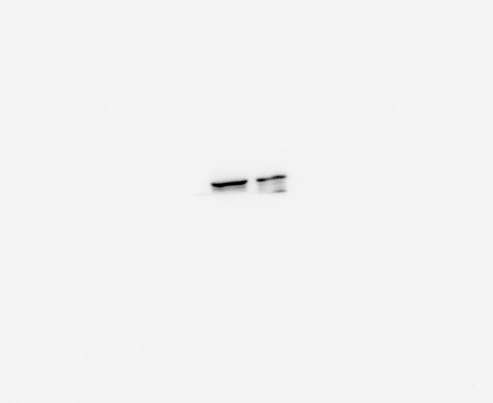

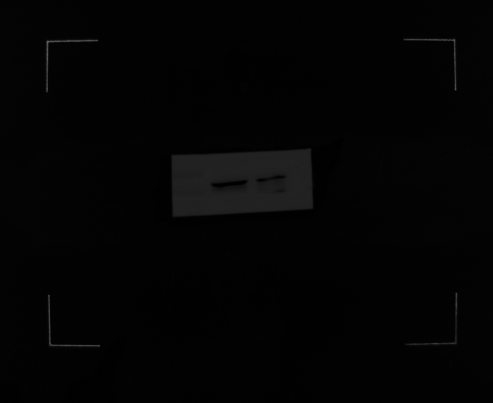


p-STING


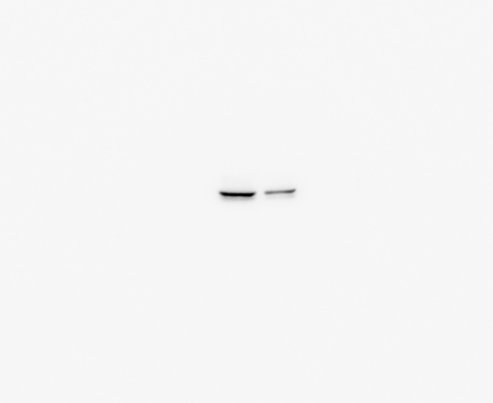

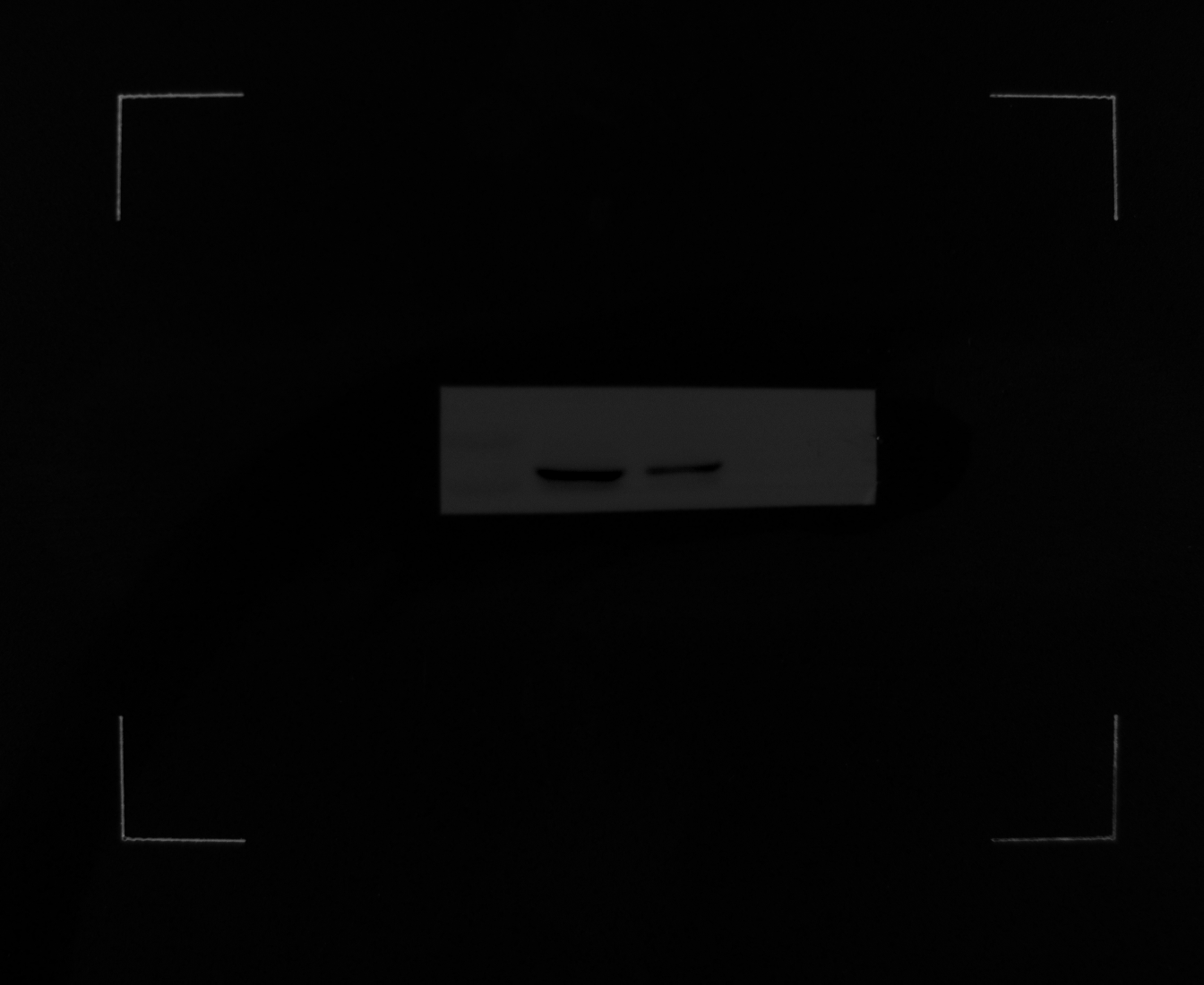


STING


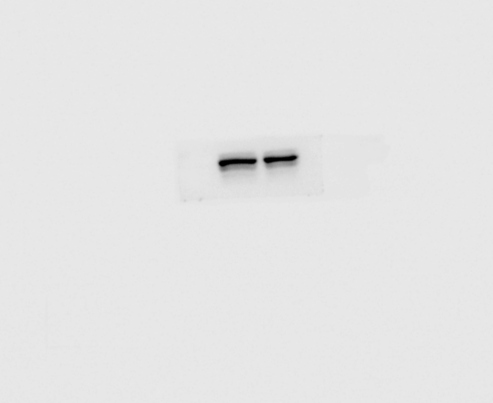

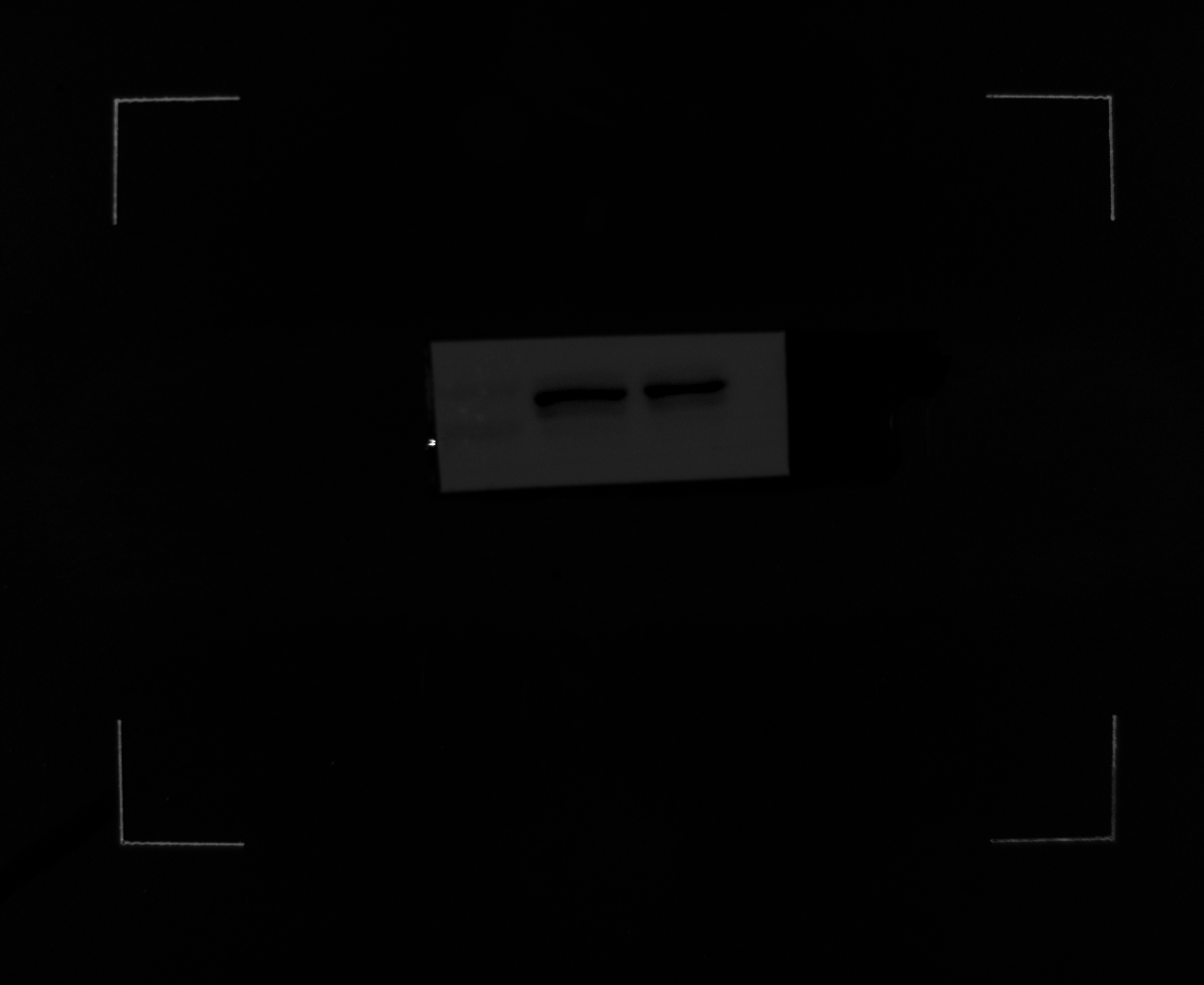


p-TBK1


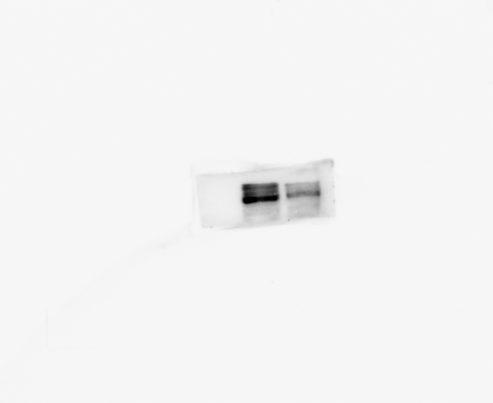

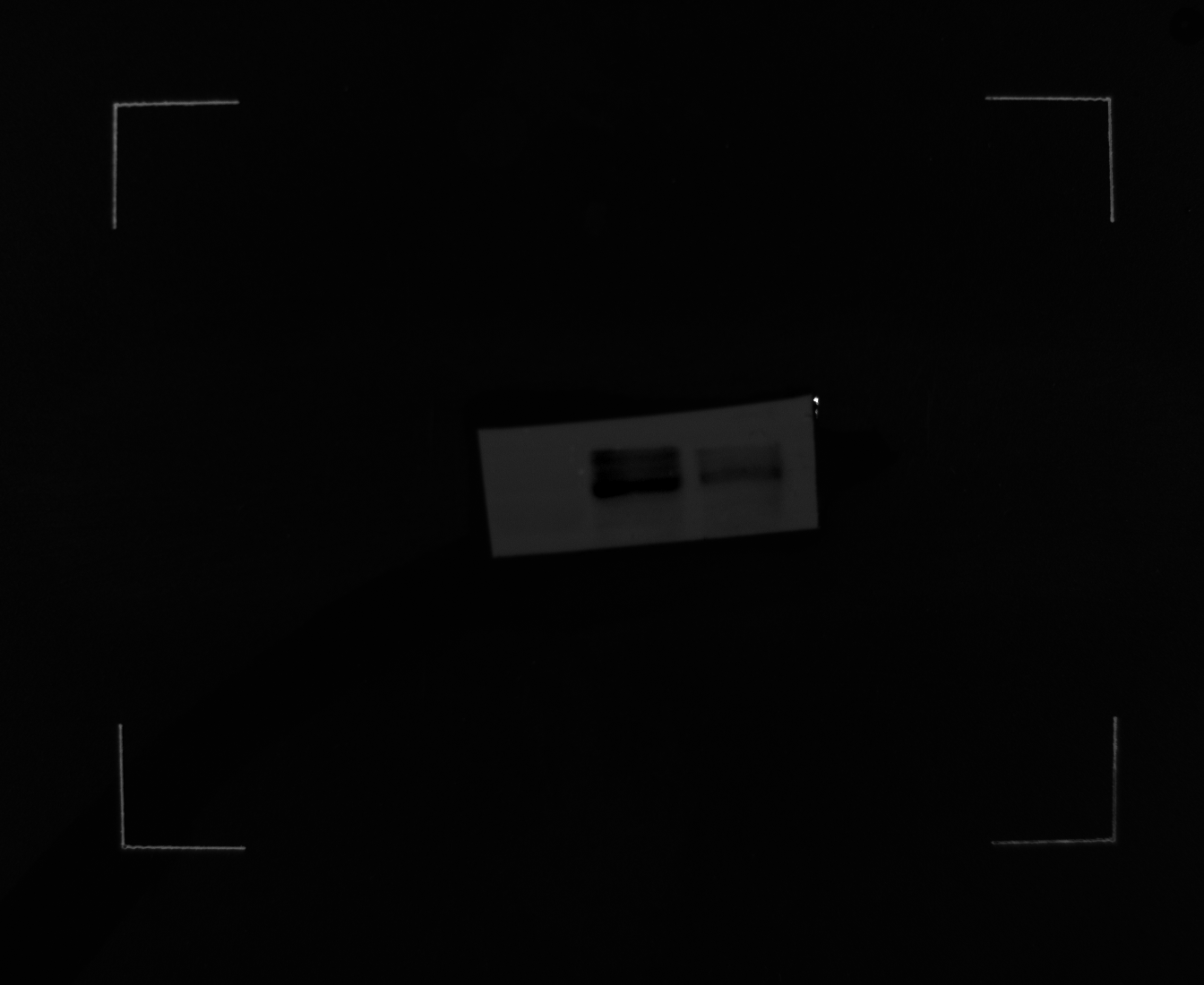


TBK1


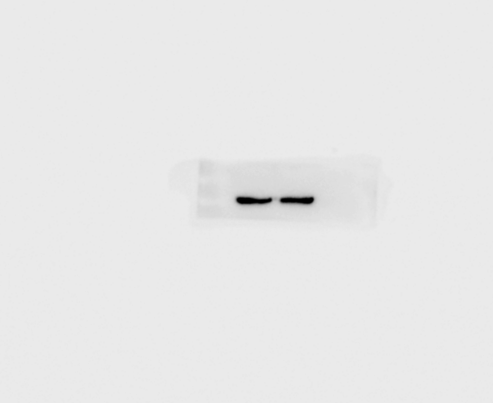

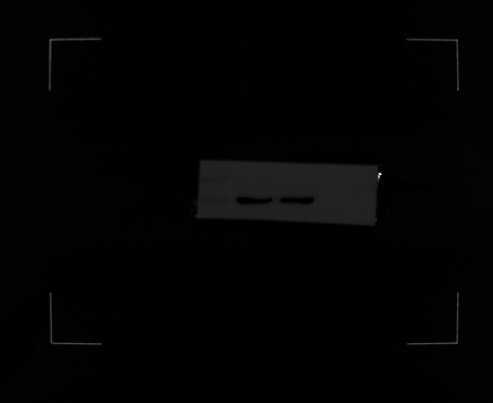


p-IRF3


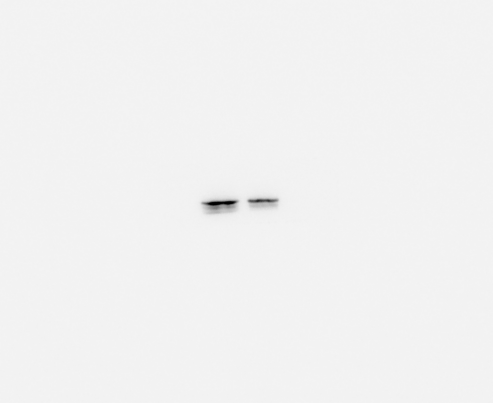

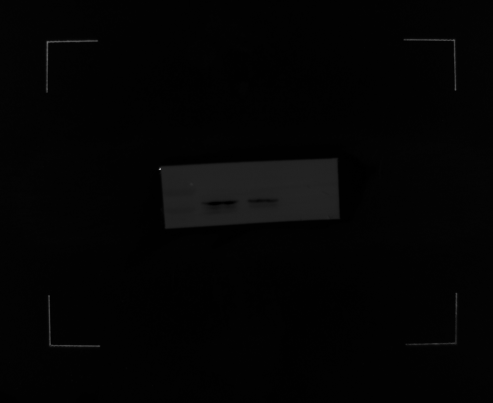


IRF3


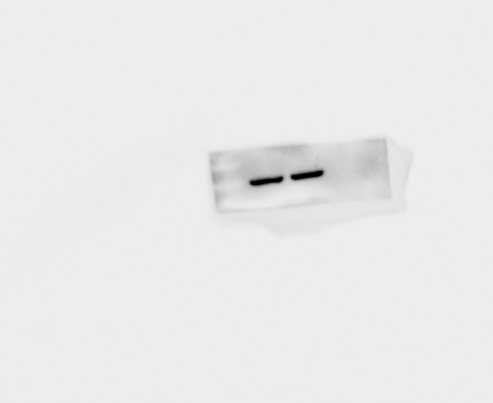

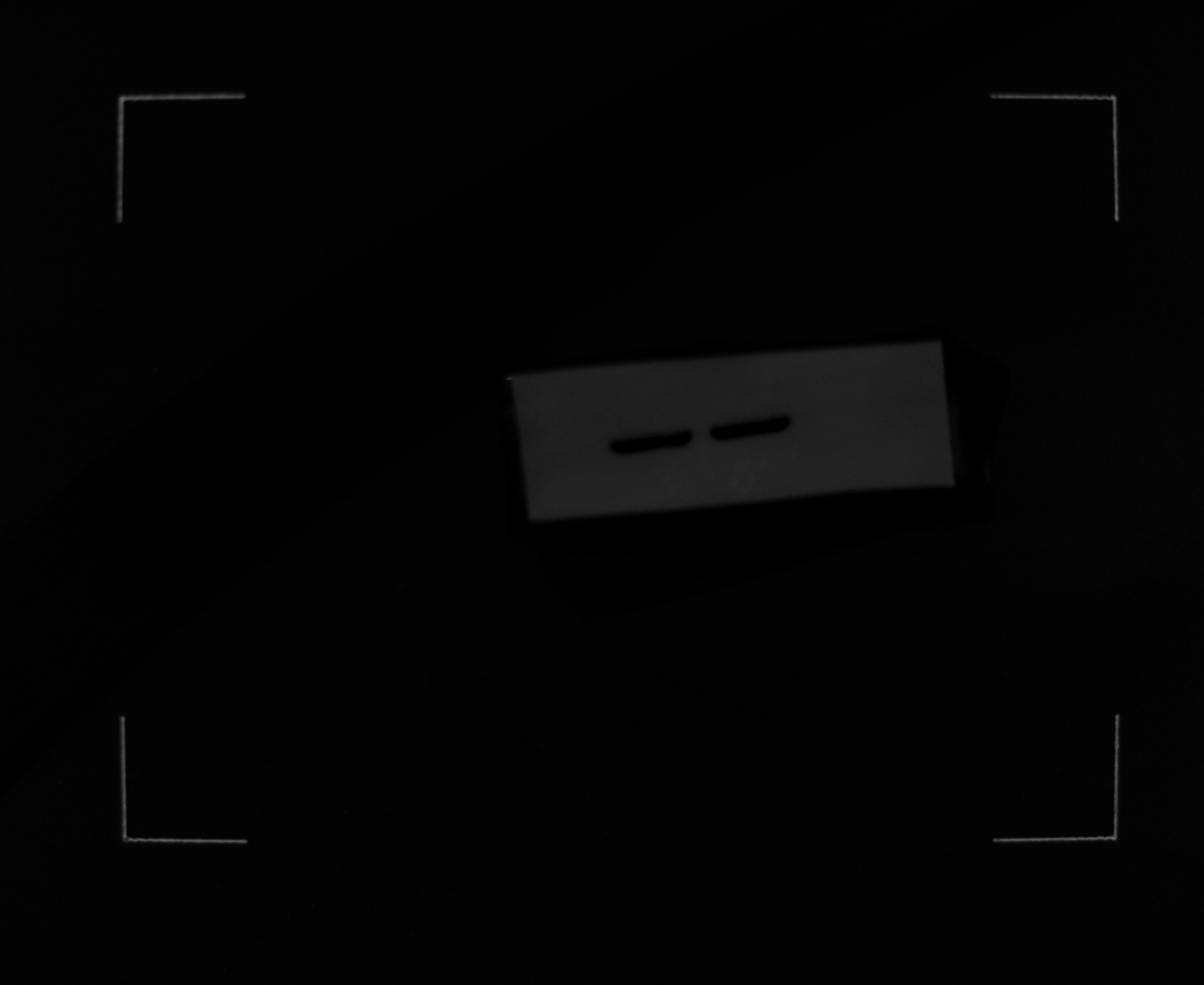


β-actin


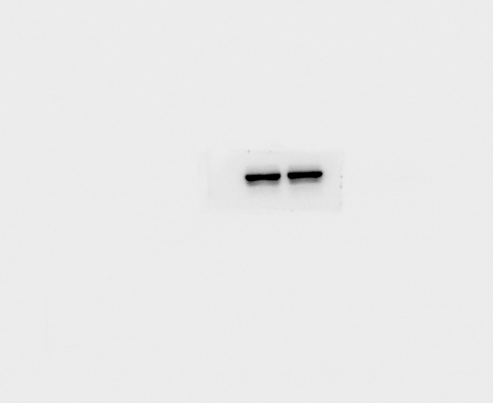

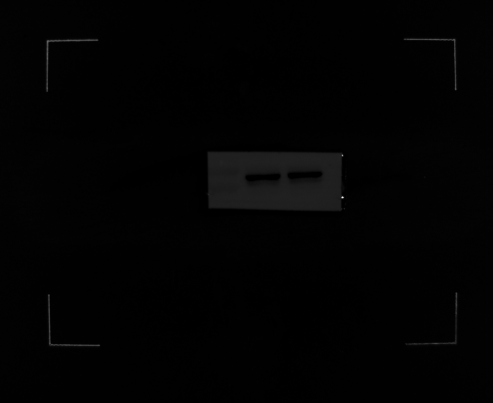


Fig 4B

cGAS


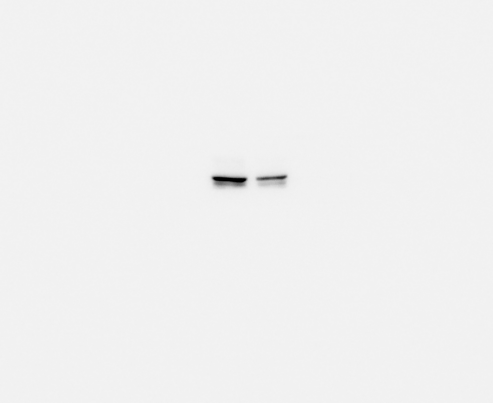

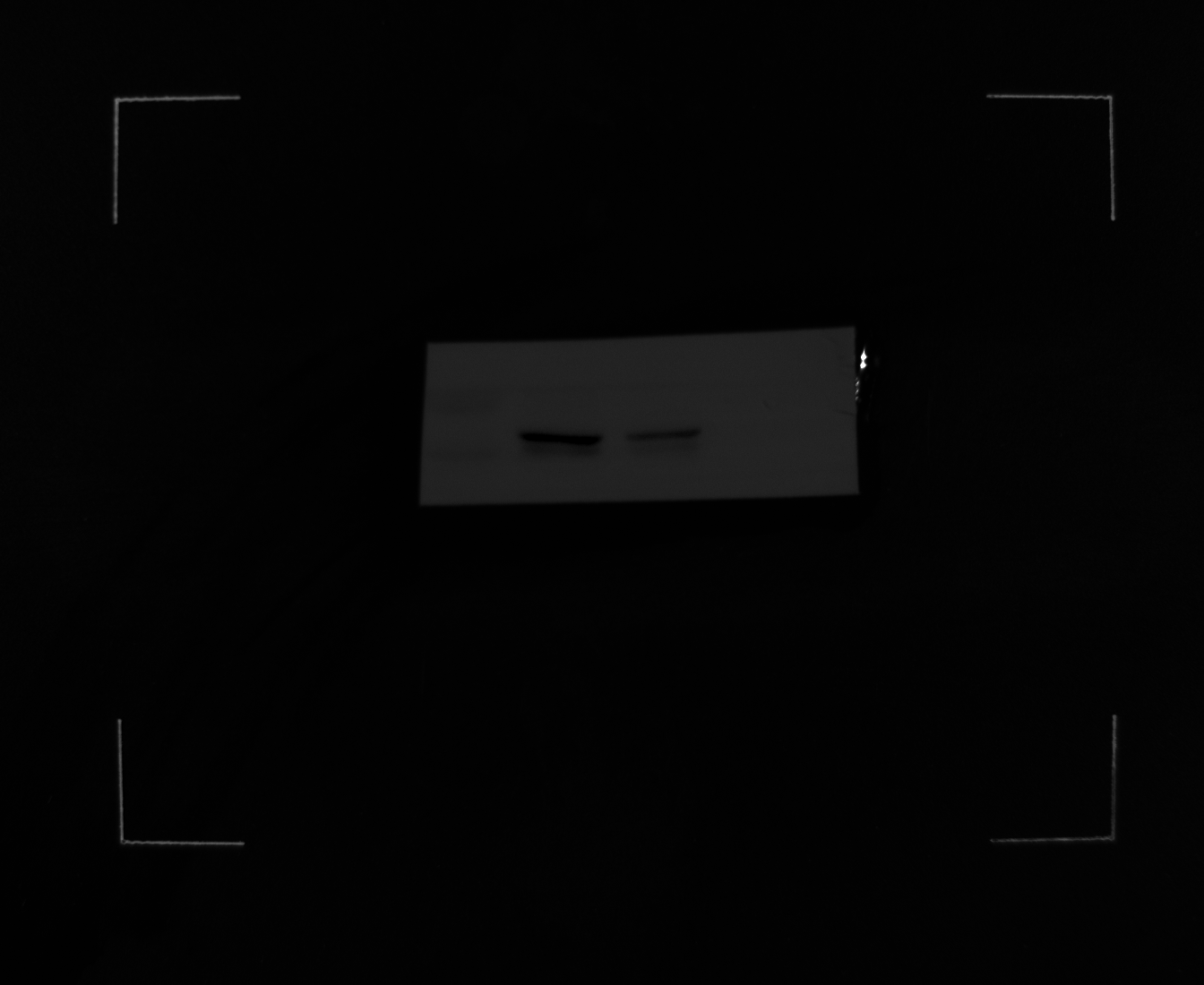


p-STING


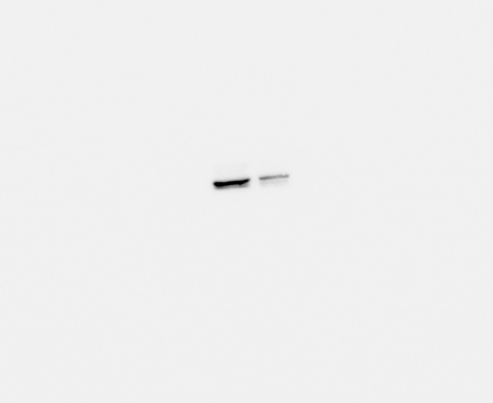

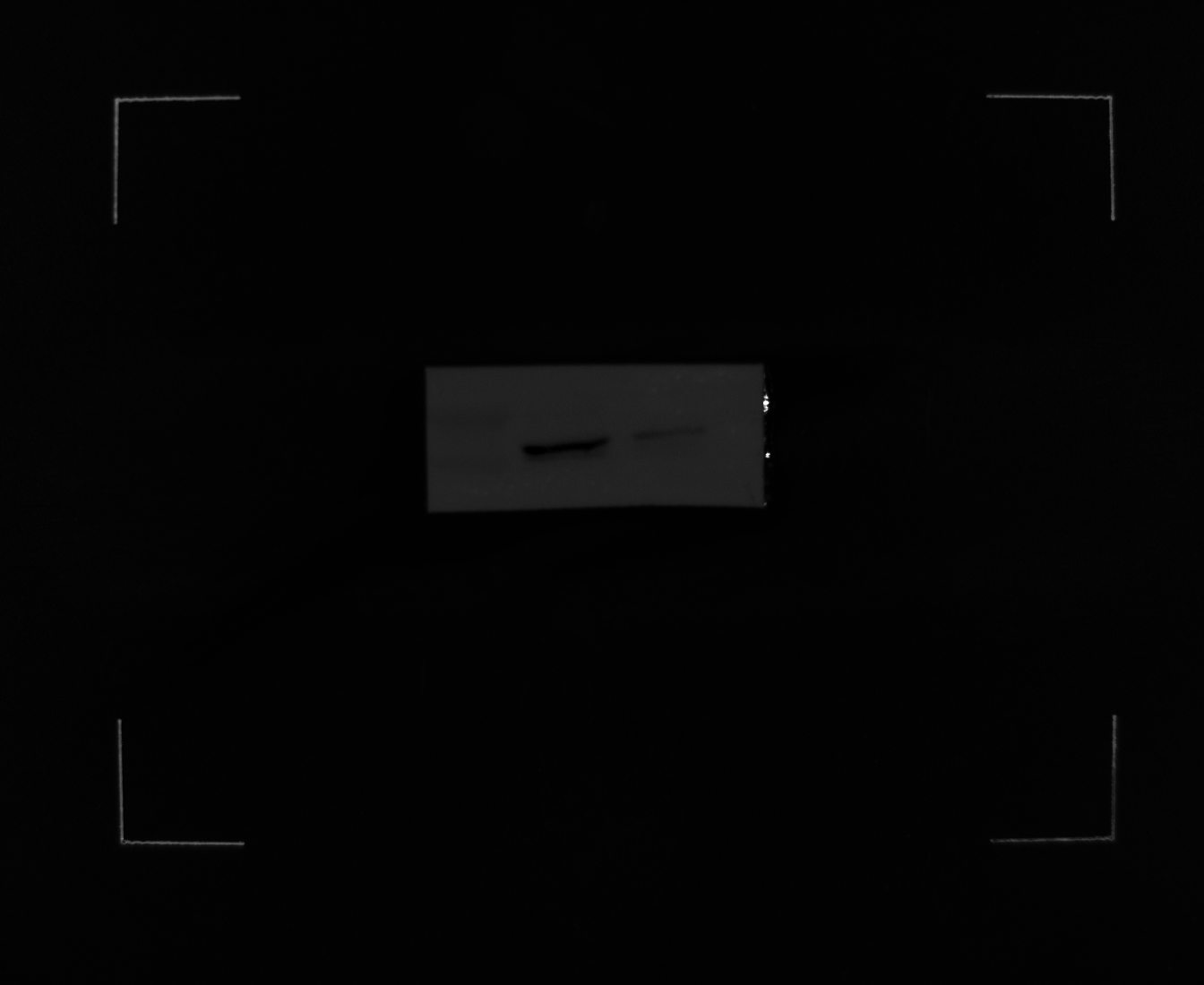


STING


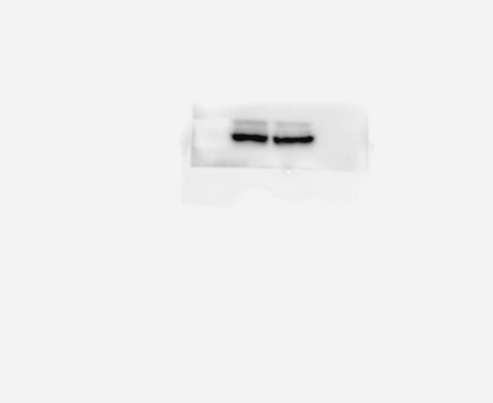

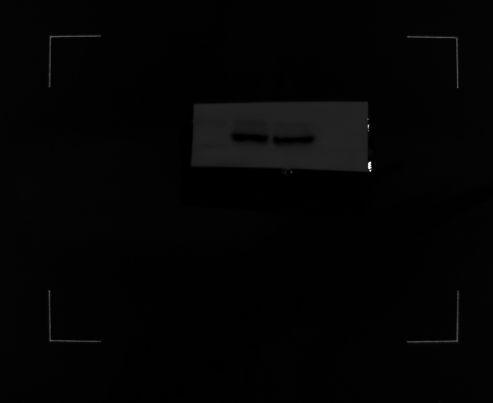


p-TBK1


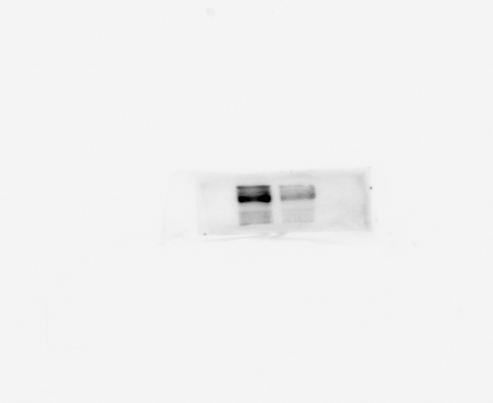

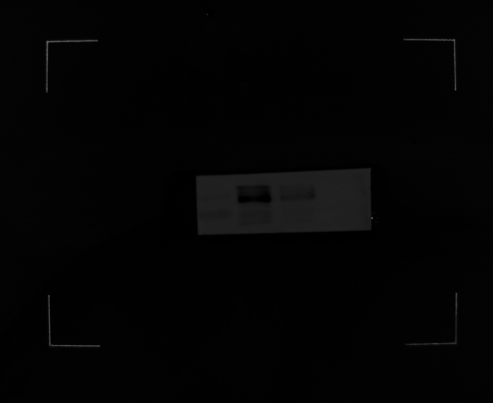


TBK1


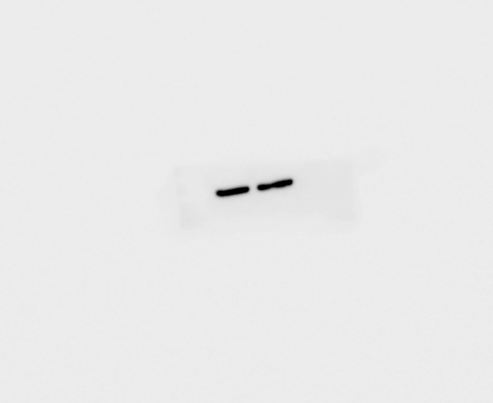

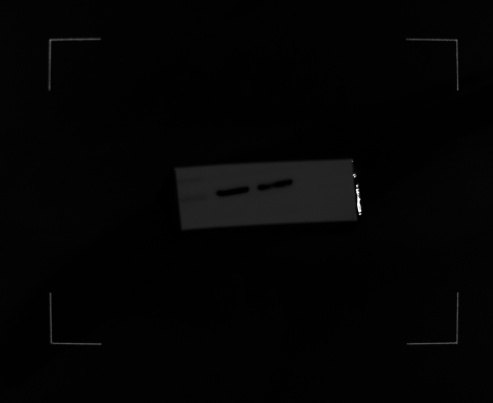


p-IRF3


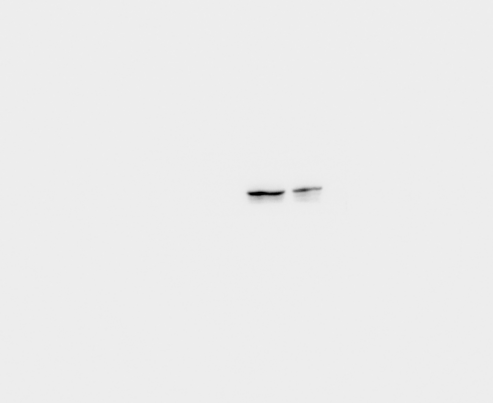

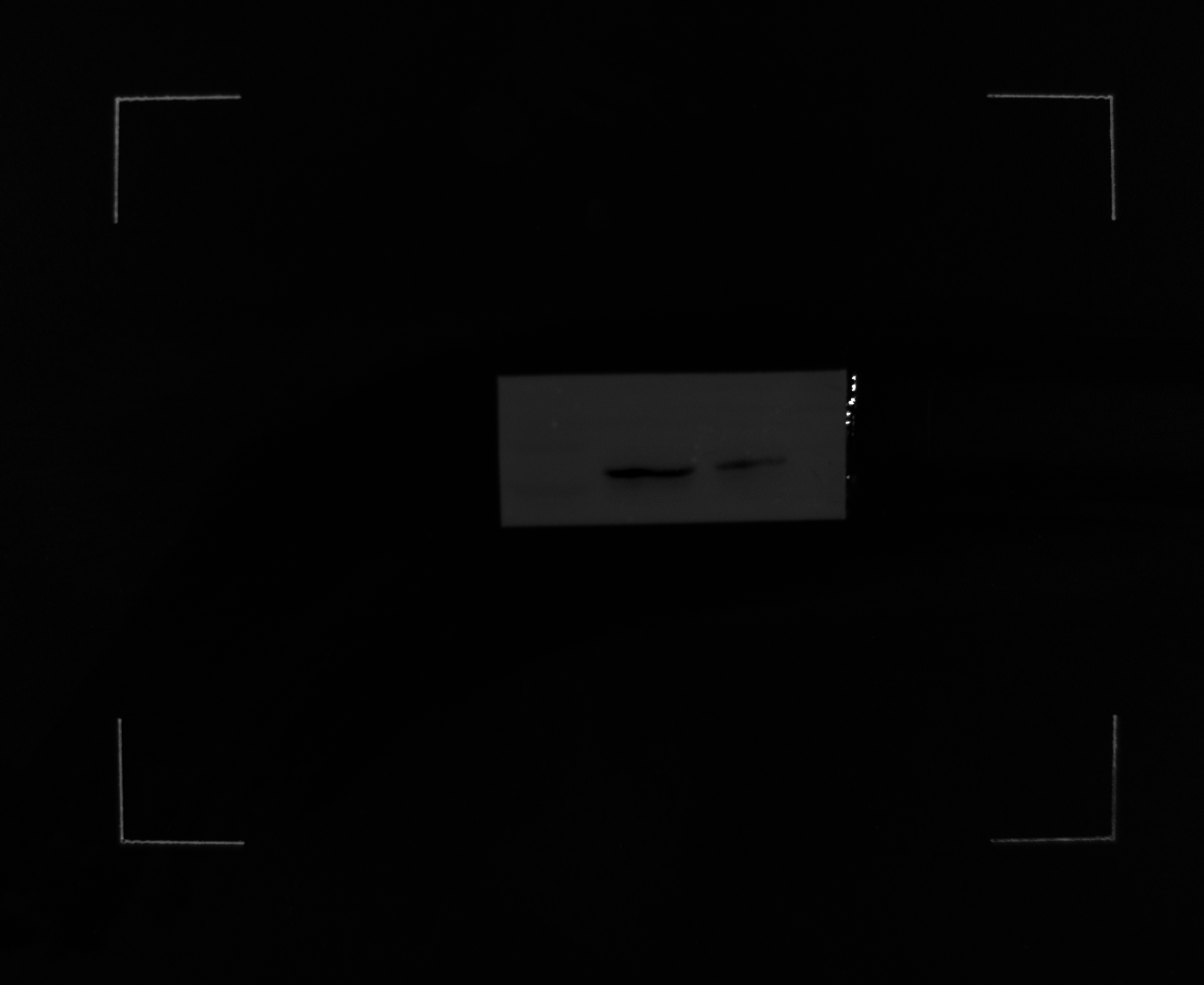


IRF3


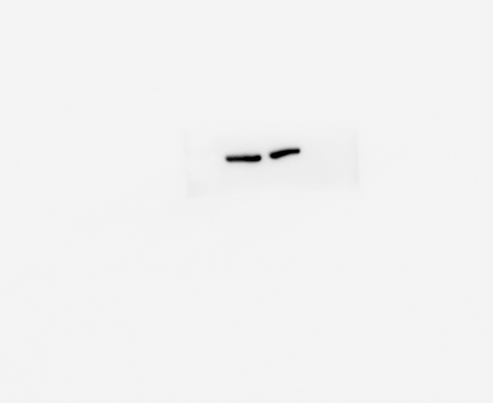

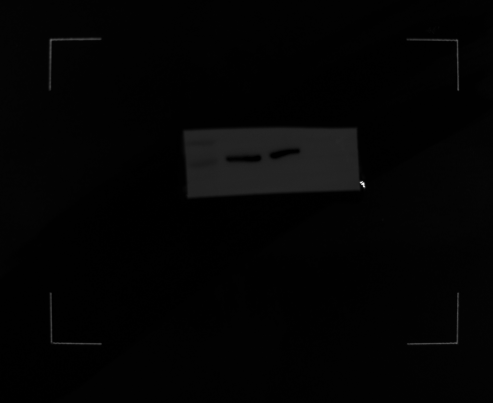


β-actin


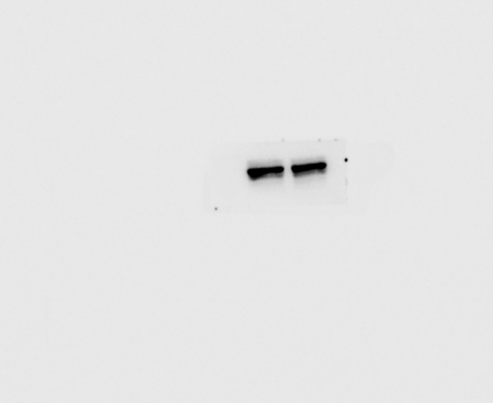

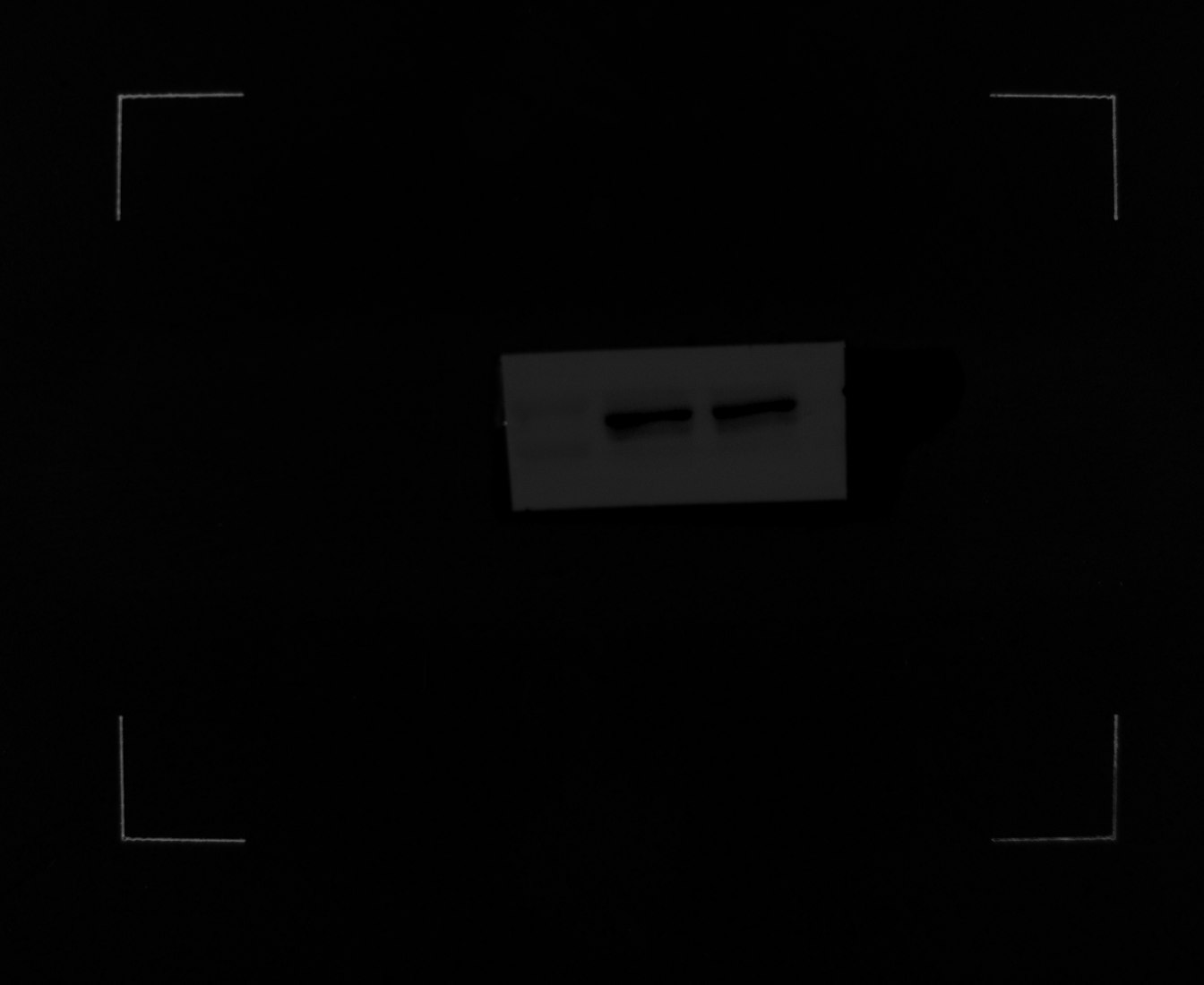

Supplement: Supplementary file 1 — Supplementary Material 1 [file 12957_2024_3487_MOESM1_ESM.docx]
