# Supplementary material for: Gut microbiota affects the activation of STING pathway and thus participates in the progression of colorectal cancer
Source: World J Surg Oncol. 2024 Jul 25;22:192. doi: 10.1186/s12957-024-03487-2 (PMC11270765; doi:10.1186/s12957-024-03487-2)
Supplement: Supplementary file 2 — Supplementary Material 2 [file 12957_2024_3487_MOESM2_ESM.pdf]

Change of authorship request form - Journals (pre-acceptance)

SPRINGER NATURE

Important information. Please read.

This form should be used by authors to request any change in authorship (adding/deleting authors) including changes in corresponding authors. This form should not be used for name changes. Please fully complete all sections. Use black ink and block capitals and provide each author's full name with the given name first followed by the family name.

By signing this declaration, all authors guarantee that the order of the authors are in accordance with their scientific contribution, if applicable as different conventions apply per discipline, and that only authors have been added who made a meaningful contribution to the work.

Please note, in author collaborations where there is formal agreement for representing the collaboration, it is sufficient for the representative or legal guarantor (usually the corresponding author) to complete and sign the Authorship Change Form on behalf of all authors, **next to the added/removed author(s)**. **(Complete Section 3, followed by Section 6.)**
In author collaborations where there is no formal agreement for representing the collaboration and **there are more than 10 authors**, one may sign for all, provided the signer appends correspondence that attests that each of the authors have agreed to the change **and the added/removed authors sign the form**. **(Complete Section 3, followed by Section 6.)**

Please note, we cannot investigate or mediate any authorship disputes. If you are unable to obtain agreement from all authors (including those who you wish to be removed) you must refer the matter to your institution(s) for investigation. Please inform us if you need to do this.

If you are not able to return a fully completed form within **30 days** of the date that it was sent to the author requesting the change, we may have to withdraw your manuscript. We cannot publish manuscripts where authorship has not been agreed by all authors (including those who have been removed).

Incomplete forms will be rejected.

Please return/upload this form, fully completed, to the Journals Editorial Office. The Journal and/or Publisher will consider the information you have provided to decide whether to approve the proposed change in authorship. We may decide to contact your institution for more information or undertake a further investigation, if appropriate, before making a final decision.

SPRINGER NATURE Springer Nature is one of the world's leading global research, educational and professional publishers, created in May 2015 through the combination of Nature Publishing Group, Palgrave Macmillan, Macmillan Education and Springer Science+Business Media.

Change of authorship request form - Journals (pre-acceptance)

SPRINGER NATURE

Section 1: Please provide the current title of manuscript

Manuscript ID no.: c66701c7-8468-45a0-a81f-f5632a1b71aa v2.0

Title: Gut microbiota affects the activation of STING pathway and thus participates in the progression of colorectal cancer

Section 2: Please provide the previous authorship, in the order shown on the manuscript before the changes were introduced. Please indicate the corresponding author by adding (CA) behind the name.

	First name(s)	Family name	ORCID or SCOPUS id, if available
1st author	Xinqiang	Liu	
2nd author	Shasha	Cui	
3rd author	Lu	Zhang	
4th author	Sainan	Wu	
5th author	Cunzhi	Feng	
6th author	Baozhi	Liu	
7th author	Jinjin	Li	
8th author	Huanlian	Yang	
9th author			
10th author			

Please use an additional sheet if there are more than 10 authors.

SPRINGER NATURE Springer Nature is one of the world's leading global research, educational and professional publishers, created in May 2015 through the combination of Nature Publishing Group, Palgrave Macmillan, Macmillan Education and Springer Science+Business Media.

Change of authorship request form - Journals (pre-acceptance)

Section 3: Please provide a justification for change. Please use this section to explain your reasons for changing the authorship of your manuscript, e.g. what necessitated the change in authorship? Please refer to the (journal) policy pages for more information about authorship. Please explain why omitted authors were not originally included and/or why authors were removed on the submitted manuscript.

Section 4: Proposed new authorship. Please provide your new authorship list in the order you would like it to appear on the manuscript. Please indicate the corresponding author by adding (CA) behind the name. <u>If the Corresponding Author has changed, please indicate the reason under section 3</u>.

	First name(s)	Family name (this name will appear in full on the final publication and will be searchable in various abstract and indexing databases)	Affiliated institute	E-mail address
1st author	Xinqiang	Liu	Binzhou People's Hospital	878374021@qq.com
2nd author	Shasha	Cui	Binzhou People's Hospital	css1985css@163.com
3rd author	Lu	Zhang	Binzhou People's Hospital	zlwp12345@sohu.com
4th author	Sainan	Wu	Binzhou People's Hospital	1752803425@qq.com
5th author	Cunzhi	Feng	Binzhou People's Hospital	79959141@qq.com
6th author	Baozhi	Liu	Binzhou People's Hospital	458649635@qq.com
7th author	Huanlian	Yang	Binzhou People's Hospital	bzliuxq1984@163.com
8th author				
9th author				
10th author				

Please use an additional sheet if there are more than 10 authors.

SPRINGER NATURE Springer Nature is one of the world's leading global research, educational and professional publishers, created in May 2015 through the combination of Nature Publishing Group, Palgrave Macmillan, Macmillan Education and Springer Science+Business Media.

SPRINGER NATURE

Change of authorship request form - Journals (pre-acceptance)

Section 5: Author contribution, Acknowledgement and Disclosures. Please use this section to provide a new disclosure statement and, if appropriate, acknowledge any contributors who have been removed as authors and ensure you state what contribution any new authors made (if applicable per the journal or book (series) policy). Please ensure these are updated in your manuscript - after approval of the change(s) - as our production department will not transfer the information in this form to your manuscript.

New acknowledgements:

This work was supported by the Binzhou People's Hospital level project (YBKTZR202116).

New Disclosures (financial and non-financial interests, funding):

This work was supported by the Binzhou People's Hospital level project (YBKTZR202116).

New Author Contributions statement (if applicable per the journal policy):

Guarantor of integrity of the entire study: Xinqiang Liu
Study concepts: Shasha Cui
Study design: Xinqiang Liu
Definition of intellectual content: Lu Zhang
Literature research: Huanlian Yang
Clinical studies: Xinqiang Liu
Experimental studies: Xinqiang Liu
Data acquisition: Lu Zhang
Data analysis: Xinqiang Liu
Statistical analysis: Sainan Wu
Manuscript editing: Xinqiang Liu
Manuscript review: Shasha Cui

State 'Not applicable' if there are no new authors.

SPRINGER NATURE Springer Nature is one of the world's leading global research, educational and professional publishers, created in May 2015 through the combination of Nature Publishing Group, Palgrave Macmillan, Macmillan Education and Springer Science+Business Media.

SPRINGER NATURE

Change of authorship request form - Journals (pre-acceptance)

Section 6: Declaration of agreement. *All* authors, unchanged, new and removed *must* sign this declaration.
(NB: Please print the form, (docu)-sign and return/upload a scanned copy. Please note that signatures that have been inserted as an image file are acceptable as long as it is handwritten. Typed names in the signature box are unacceptable.) *** Please delete as appropriate. Delete all of the bold if you were on the original authorship list and are remaining as an author.**

	First name	Family name		Signature	Date
1st author	Xinqiang	Liu	I agree to the proposed new authorship shown in section 4	刘新强 Xingiang Liu	13/06/2024
2nd author	Shasha	Cui	I agree to the proposed new authorship shown in section 4	崔莎莎 Shasha Cui	13/06/2024
3rd author	Lu	Zhang	I agree to the proposed new authorship shown in section 4	张禄 Lu zhang	13/06/2024
4th authors	Sainan	Wu	I agree to the proposed new authorship shown in section 4	吴赛男 Sainan Wu	13/06/2024
5th author	Cunzhi	Feng	I agree to the proposed new authorship shown in section 4	封存志 Cunzhi Feng	13/06/2024
6th author	Baozhi	Liu	I agree to the proposed new authorship shown in section 4	刘宝志 Baozhi Liu	13/06/2024
7th author	Jinjin	Li	I agree to the proposed new authorship shown in section 4 **and the removal of my name to the authorship list**	李金金 Jinjin Li	13/06/2024

SPRINGER NATURE Springer Nature is one of the world's leading global research, educational and professional publishers, created in May 2015 through the combination of Nature Publishing Group, Palgrave Macmillan, Macmillan Education and Springer Science+Business Media.

SPRINGER NATURE

Change of authorship request form - Journals (pre-acceptance)

	First name	Family name		Signature	Date
8th author	Huanlian	Yang	I agree to the proposed new authorship shown in section 4	杨焕莲 HuanLian Yang	13/06/2024
9th author			I agree to the proposed new authorship shown in section 4 /**and the addition/removal*of my name to the authorship list** /and the proposed change in corresponding author		
10th author			I agree to the proposed new authorship shown in section 4 /**and the addition/removal*of my name to the authorship list** /and the proposed change in corresponding author		

Please use an additional sheet if there are more than 10 authors.

In case of author collaborations with formal agreement:

	Name of consortium/consortia	First name	Family name		Signature	Date
Representative/legal guarantor				I agree to the proposed new authorship shown in section 4 /**and the addition/removal*of my name to the authorship list** /and the proposed change in corresponding author		

Both added/removed authors should complete the information in the first table under Section 6.

---- End of form ----

SPRINGER NATURE Springer Nature is one of the world's leading global research, educational and professional publishers, created in May 2015 through the combination of Nature Publishing Group, Palgrave Macmillan, Macmillan Education and Springer Science+Business Media.
